# Association of Gut Microbiota with Atherogenic Dyslipidemia, and Its Impact on Serum Lipid Levels after Bariatric Surgery

**DOI:** 10.3390/nu14173545

**Published:** 2022-08-28

**Authors:** Priscilla López-Montoya, Daniel Cerqueda-García, Marcela Rodríguez-Flores, Blanca López-Contreras, Hugo Villamil-Ramírez, Sofía Morán-Ramos, Selene Molina-Cruz, Berenice Rivera-Paredez, Bárbara Antuna-Puente, Rafael Velázquez-Cruz, Teresa Villarreal-Molina, Samuel Canizales-Quinteros

**Affiliations:** 1 Unidad de Genómica de Poblaciones Aplicada a la Salud, Facultad de Química, Universidad Nacional Autónoma de México (UNAM), Mexico City 14610, Mexico; prisc_lopezm@hotmail.com (P.L.-M.); dacegabiol@ciencias.unam.mx (D.C.-G.); blopez@inmegen.gob.mx (B.L.-C.); hugovira@unam.mx (H.V.-R.); smoran@inmegen.gob.mx (S.M.-R.); selenemolinacruz@gmail.com (S.M.-C.); 2 Instituto Nacional de Medicina Genómica (INMEGEN), Mexico City 14610, Mexico; 3 Programa de Maestría en Ciencias Bioquímicas, Facultad de Química, Universidad Nacional Autónoma de México (UNAM), Mexico City 04510, Mexico; 4 Unidad de Investigación de Enfermedades Metabólicas, Instituto Nacional de Ciencias Médicas y Nutrición Salvador Zubirán, Mexico City 14080, Mexico; chelorf76@yahoo.com; 5 Programa de Doctorado en Ciencias Biomédicas, Universidad Nacional Autónoma de México (UNAM), Mexico City 04510, Mexico; 6 Centro de Investigación en Políticas, Población y Salud (CIPPS), Facultad de Medicina-UNAM, Mexico City 04510, Mexico; bereriveraparedez7@gmail.com; 7 Infection Disease Division, Department of Medicine, Queen’s University, Kingston, ON K7L3N6, Canada; bapuente@gmail.com; 8 Laboratorio de Genómica del Metabolismo Óseo, INMEGEN, Mexico City 14610, Mexico; rvelazquez@inmegen.gob.mx; 9 Laboratorio de Enfermedades Cardiovasculares, INMEGEN, Mexico City 14610, Mexico; mvillareal@inmegen.gob.mx

**Keywords:** gut microbiota, dyslipidemia, HDL-C, triglycerides, 16S rRNA

## Abstract

Gut microbiota has been suggested to modulate circulating lipids. However, the relationship between the gut microbiota and atherogenic dyslipidemia (AD), defined as the presence of both low HDL-C and hypertriglyceridemia, is not fully understood. Moreover, because obesity is among the main causes of secondary AD, it is important to analyze the effect of gut microbiota composition on lipid profiles after a weight loss intervention. We compared the microbial diversity and taxonomic composition in patients with AD (*n* = 41) and controls (*n* = 38) and sought correlations of genera abundance with serum lipid levels in 20 patients after weight loss induced by Roux-en-Y gastric bypass (RYGB) surgery. Gut microbiota composition was profiled using next-generation sequencing of 16S rRNA. Gut microbiota diversity was significantly lower in atherogenic dyslipidemia. Moreover, relative abundance of two genera with LDA score >3.5 (*Megasphaera* and LPS-producing *Escherichia-Shigella*), was significantly higher in AD subjects, while the abundance of four short chain fatty acids (SCFA) producing-genera (*Christensenellaceae R-7*, *Ruminococcaceae UCG-014*; *Akkermansia* and *[Eubacterium] eligens group*) was significantly higher in controls. Notably, *[Eubacterium] eligens group* abundance was also significantly associated with higher HDL-C levels in RYGB patients one year after surgery. Although dietary polyunsaturated fatty acid/saturated fatty acid (PUFA/SFA) ratio and PUFA intake were higher in controls than in AD subjects, of the four genera differentiated in cases and controls, only *Akkermansia* abundance showed a positive and significant correlation with PUFA/SFA ratio. Our results suggest that SCFA-producing bacteria promote a healthy lipid homeostasis, while the presence of LPS-producing bacteria such *Escherichia-Shigella* may contribute to the development of atherogenic dyslipidemia.

## 1. Introduction

Dyslipidemia characterized by elevated triglyceride (TG), low HDL cholesterol (HDL-C), and high LDL cholesterol (LDL-C) plasma concentrations are among the main risk factors for cardiovascular disease (CVD) [1,2]. The prevalence of these dyslipidemias varies among ethnic groups and Mexican Americans show a higher prevalence of elevated TG and low HDL-C levels as compared to other ethnic groups in the USA [3]. Low HDL-C levels are a well-known independent epidemiological risk factor for CVD, and this dyslipidemia is highly prevalent in Mexican adults [4,5,6]. Moreover, the combination of low HDL-C and elevated triglyceride levels is considered as atherogenic dyslipidemia (AD) [7,8].

Among the various lifestyle factors affecting serum lipid levels, habitual intake of nutrients such as dietary fat and unsaturated/saturated fatty acids is closely related to lipid metabolism [9,10], and has been recognized as an important determinant in the development of dyslipidemia [11]. Moreover, it has recently become clear that the gut microbiota contributes to host metabolism regulation, including circulating lipid levels [12,13,14,15,16]. Gut microbiota was found to explain 6% variance of triglyceride and 4% of variance of HDL-C serum levels but has a small effect on LDL-C and total cholesterol levels [12]. Gut microbiota produces enzymes involved in dietary lipid and bile acid metabolism that affect the blood lipid profile of the host. Moreover, gut microbiota may modulate lipid absorption, potentially altering intestinal lipoprotein formation [17]. However, how the plasma lipid profile is modified by the gut microbiota to date is not fully understood.

There are few studies seeking associations between gut microbiota and lipid concentrations in humans, which have consistently reported that lower gut microbiota alpha-diversity is associated with higher triglyceride and lower HDL-C levels. Although several genera have been associated with lipid levels, these associations have been inconsistent [12,16,18]. Furthermore, because certain bacterial genera vary in abundance among different ethnicities, it is important to seek these associations in populations with a high prevalence of dyslipidemias, such as the Mexican population.

Obesity is intimately associated with the development of dyslipidemia [19,20]. Different weight loss interventions are known to improve triglyceride and HDL-C levels [21,22]. Notably, patients undergoing Roux-en-Y gastric bypass (RYGB) surgery show significant changes in the gut microbiota along with improved lipid levels [23]. However, whether the post-RYGB gut microbiota profile is associated with improvement of lipid levels after bariatric surgery requires further study.

Thus, we analyzed case–control associations of gut microbiota with atherogenic dyslipidemia (low HDL-C and hypertriglyceridemia), and its effect on serum lipid levels after bariatric surgery.

## 2. Materials and Methods

### 2.1. Study Populations

We studied 41 subjects with AD, and 38 controls aged 18 or older recruited as part of the Health Workers Cohort Study and at the Hospital Infantil de México Federico Gómez in Mexico City. Participants were recruited as described elsewhere [24,25]. Exclusion criteria included the use of antibiotics three months before recruitment or the use of lipid lowering drugs in subjects without a diagnosis of dyslipidemia. Additionally, 20 patients with obesity undergoing laparoscopic RYGB were recruited from the Bariatric Surgery Program at the Instituto Nacional de Ciencias Médicas y Nutrición in Mexico City.

The project was approved by the Ethics Committees of participant Institutions, and all participants signed informed consent.

For patients with AD and controls, anthropometric parameters such as weight, height, waist and hip circumferences were measured by trained personnel following standardized procedures and calibrated equipment. Body mass index (BMI) was calculated as weight (kg) divided by squared height (meters) and obesity was considered as BMI ≥30 kg/m^2^ according to the World Health Organization (WHO) classification (WHO, 2021). For patients undergoing bariatric surgery, all measurements were taken by nutritionists of a multidisciplinary team, before and 12 months after the intervention.

### 2.2. Habitual Dietary Intake Assessment

A semi-quantitative food frequency questionnaire (FFQ) previously validated in the Mexican population [26] was used to evaluate habitual dietary intake. This questionnaire assesses the consumption of 116 food items during the previous year. For calculating average daily energy and nutrient intake, data from the FFQ were captured and computed through the Evaluation System of Nutritional Habits and Nutrient Intake software [27]. Daily consumed grams of proteins, carbohydrates and different types of fats such as monounsaturated fatty acids (MUFAs), polyunsaturated fatty acids (PUFAs) and saturated fatty acids (SFAs) were converted to kilocalories using the corresponding Atwater factor [28]. In this way, consumption of each macronutrient was expressed as the percentage of the daily energy intake. The total dietary fiber intake in grams was standardized per 1000 kilocalories to reduce inter-individual energy intake variation.

### 2.3. Biochemical Determinations

Blood samples were drawn after 8–12 h of overnight fasting to determine serum levels of glucose, total cholesterol, triglycerides, and HDL-C. Atherogenic dyslipidemia was defined as the simultaneous presence of elevated levels of triglycerides ≥150 mg/dL and low HDL-C (high-density lipoprotein cholesterol) <40 mg/dL in men and <45 mg/dL in women [29]. Type 2 diabetes was defined considering a fasting glucose measurement ≥126 mg/dL or previous self-reported diagnosis [30].

### 2.4. Stool Sampling

Fecal samples were collected at home in a sterile polypropylene container following printed instructions to avoid contamination. Aliquots of 180–220 mg were stored at −70 °C until processing. DNA was extracted from aliquots using QIAamp^®^ DNA Stool or Power fecal kit (Qiagen, Hilden, Germany.) adding a previous step of mechanical sample lysis with a FastPrep device. The final elution volume was 200 µL and was stored at −20 °C until further analysis. DNA concentration and purity were determined by spectrophotometry (Nanodrop 2000c, Thermo Scientific, Wilmington, DE, USA).

### 2.5. 16S rRNA Sequencing

DNA samples from 30 AD patients and 31 controls were sequenced using the primers 515F and 806R of the 16S rRNA gene V4 hypervariable region as described elsewhere [31]. Samples from the remaining 11 AD cases, 7 controls and the 20 RYGB patients before and 12 months after surgery were sequenced to amplify the 16S rRNA gene V3-V4 region, following the protocol for Illumina library preparation. Briefly, a first PCR was run using the primers with attached overhang adapters. The amplicons were purified using AMPure XP beads (Beckman Coulter). A second PCR was executed employing the Nextera XT Index Kit (Illumina) to incorporate dual indices and the Illumina sequencing adapters. The resulting libraries were purified with AMPure XP beads, amplicon size and concentrations were assessed with an Agilent D1000 ScreenTape for 4200 TapeStation System (Agilent Technologies) and a Qubit 2.0 fluorometer (Invitrogen), respectively. Both sequencing protocols were carried out at the Sequencing Unit of the National Institute of Genomic Medicine (INMEGEN) using the Illumina MiSeq platform.

### 2.6. Sequence Processing

The paired-end raw reads were processed using the QIIME2 pipeline [32]. Forward reads of the V3-V4 region were trimmed at position 194 in the 5′ and reverse reads were trimmed at position 20 in the 5′, with truncation at position 240 in the 3′. Forward V4 region reads were trimmed at position 20 in the 5′, the reverse reads at position 38 in the 5′, with truncation at position 220 in the 3′. A 220 bp segment was shared by reads obtained from the V3-V4 and V4 sequence protocols and was used for all analyses. The DADA2 plugin [33] was used for error correction and resolution of the amplicon sequence variants (ASVs), chimeric sequences were removed with the “consensus” method. After resolution, ASVs were grouped in operational taxonomic units (OTUs) at 97% identity using the “cluster-features-open-reference” plugin with the V-SEARCH algorithm [34] and the SILVA database (v.132). A phylogenetic tree was built with OTU representative sequences using the “align-to.three-mafft-fasttree” plugin [35,36]. Thereafter, samples were standardized by rarefaction at a 19,000 high-quality read depth, with a total of 2,261,000 reads.

### 2.7. Bioinformatic Analysis

The OTU abundance table and phylogeny tree were exported to the R (v. 4.1.1) environment for further analysis with the phyloseq package (v1.38.0) [37]. Alpha diversity was evaluated with the number of observed OTUs, Chao1, Shannon and Simpson indices. Weighted and unweighted UniFrac distance metrics were used to estimate beta diversity. A permutational multivariate analysis of variance (PERMANOVA) was used to test differences in beta diversity between groups, using the Vegan package (v2.5.7) and applying the adonis function and 9999 permutations [38].

Microbial composition differences between AD cases and controls from the phylum to genus level groups were assessed by Linear discriminant analysis Effect Size (LEfSe v1.0) [39]. A LDA score >2.0 and *p* < 0.05 was considered statistically significant. The possible effect of age, sex and BMI as covariates was assessed using multivariate linear models (MaAsLin2 v1.4.0) [40]. Analyses in MaAsLin2 were performed using default parameters. Predicted functional microbiota profiling was achieved using PICRUSt2 (Phylogenetic Investigation of Communities by Reconstruction of Unobserved States; Version 2.2.0) [41]. The metabolic pathways were annotated by MetaCyc database [42], and differences between AD and controls were assessed using LEfSe v1.0. Metabolic pathways with LDA score > 2.0 and *p* < 0.05 were considered as statistically significant. All plots were created using R (v4.1.1).

### 2.8. Statistical Analysis

Shapiro–Wilk and Levene tests were used to verify normal distribution of the data. The Mann–Whitney U test was conducted to compare non-normally distributed variables between AD patients and controls and the Wilcoxon signed rank test to compare variables before and after RYGB surgery. A two-tailed Student’s *t*-test was used to compare diversity indices, and *X*^2^ test was used to compare categorical variables. These analyses were performed using SPSS (version 24.0, SPSS Inc., Chicago, IL, USA) and R (version 4.1.1). Statistical significance was considered when *p* < 0.05. Spearman correlations between relative genera abundance, BMI, HDL-C and triglyceride serum levels, PUFA/SFA ratio and nutrient intake were tested in R (version 4.1.1). For bariatric surgery analyses, only genera with a relative abundance >1% in at least 25% of participants were analyzed. Pearson partial correlation analyses were implemented adjusting for BMI. *p*-values were corrected for multiple testing using the Benjamini–Hochberg method [43].

## 3. Results

As expected, individuals with atherogenic dyslipidemia showed higher TG and lower HDL-C serum levels compared to control group. Moreover, the AD group showed higher BMI and lower total cholesterol serum levels (Table 1).

### 3.1. Dietary Patterns in the Atherogenic Dyslipidemia and Control Groups

**Figure 1:**
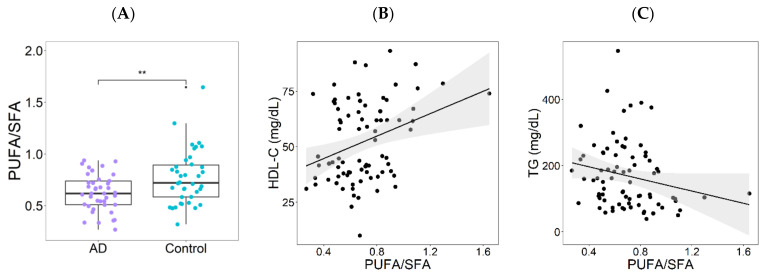
PUFA/SFA ratio and atherogenic dyslipidemia (AD). (**A**) Median PUFA/SFA ratio in study groups. (**B**) Spearman correlation between HDL-C serum levels and PUFA/SFA ratio including the whole study population (Rho = 0.256; *p* = 0.023). (**C**) Spearman correlation between TG serum levels and PUFA/SFA ratio (Rho = −0.240; *p* = 0.033). PUFA, polyunsaturated fatty acids; SFA, saturated fatty acids; HDL-C, high density lipoprotein -cholesterol; TG triglycerides. ** *p* < 0.005.

Median dietary carbohydrate percentage in AD individuals was slightly higher, while median dietary fat percentage was lower than macronutrient proportion recommendations for a healthy diet in both groups [44]. Differences in macronutrient proportions between groups were not significant. Notably, the only significant difference in nutrient intake identified between groups was higher proportion of polyunsaturated fatty acids (PUFA) intake in controls (Supplementary Table S1). PUFA/SFA ratio was significantly lower in individuals with atherogenic dyslipidemia as compared to the control group (Figure 1A). Moreover, in the whole population PUFA/SFA ratio showed a positive correlation with HDL-C levels (Rho = 0.256, *p* = 0.023) and a negative correlation with triglyceride serum levels (Rho = −0.240, *p* = 0.033; Figure 1B,C).

### 3.2. Differences in Gut Microbiota Diversity

**Figure 2:**
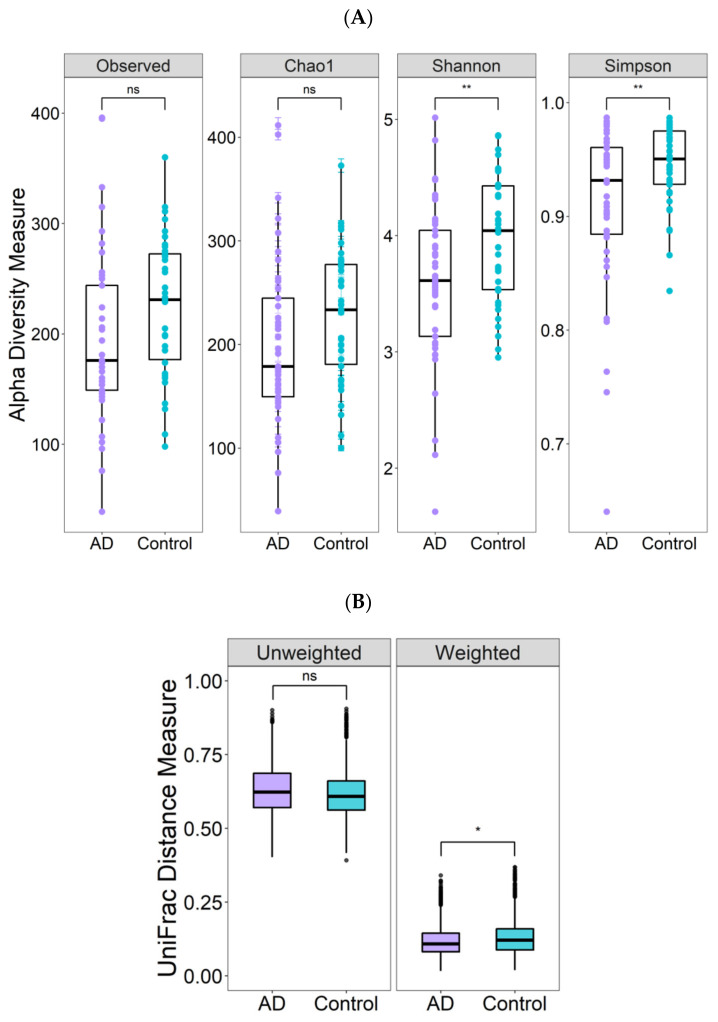
Comparison of gut microbiota diversity in atherogenic dyslipidemia (AD) patients and controls. (**A**) Alpha diversity estimates: Observed OTUs, Chao1, Shannon and Simpson indices; the plotted data represent medians and interquartile ranges. (**B**) Beta diversity estimates; the plotted data represent the weighted (F-value= 2.298; R-value= 0.0289) and unweighted (F-value= 1.299; R-value= 0.017) UniFrac distances; *p*-value was obtained using a permutational multivariate analysis of variance (PERMANOVA). * *p* < 0.05; ** *p* < 005; ns, not significant.

Alpha diversity estimated by the number of observed species, Chao1, Shannon and Simpson indices was lower in the AD group, although only the comparisons of the Shannon and Simpson indices were statistically significant (Figure 2A; *p* < 0.005). Regarding beta-diversity, significant differences between AD individuals and controls were observed only for the weighted Unifrac distance (*p* < 0.05, Figure 2B).

### 3.3. Taxonomic Gut Microbiota Differences

The average relative abundance of the gut microbiota at the phylum, class and genus levels in AD patients and controls is shown in Supplementary Figures S1–S3. The most abundant phyla in AD subjects and controls were Firmicutes (46.7% and 53.0%, respectively) and Bacteroidetes (43.5% and 37.2%, respectively); the most abundant classes were Clostridia (40.2% in AD and 48.5% in controls) and Bacteroidia (43.5% in AD and 37.2% in controls); while the most abundant genera were *Bacteroides* (20.9% in AD and 18.0% in controls) and *Prevotella 9* (13.7% in AD and 11.3% in controls).

**Figure 3:**
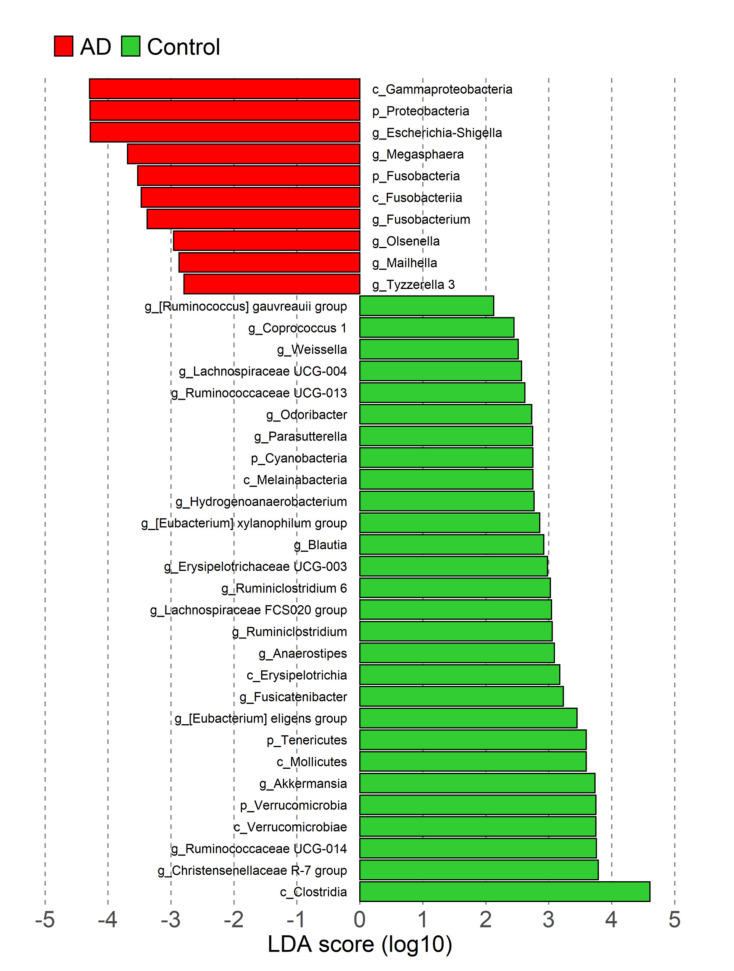
LEfSe plot showing differentially abundant phyla (p), classes (c) and genera (g) between controls (green) and atherogenic dyslipidemia (AD) subjects (red). LDA score > 2.0 and *p* < 0.05 indicate statistically significant differences.

**Figure 4:**
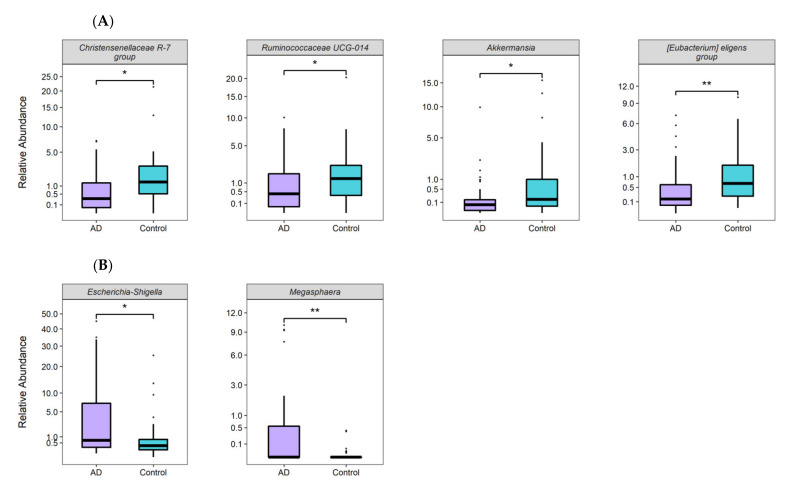
Relative abundance of differentiated genera (LDA score >3.5; *p*-value < 0.05) between AD individuals and controls. (**A**) Bacterial genera significantly more abundant in controls; (**B**) bacterial genera significantly more abundant in subjects with AD. * *p* < 0.05; ** *p* < 005.

LEfSe analysis revealed that the relative abundance of five of the ten most abundant phyla showed significant differences between AD patients and controls, Proteobateria and Fusobacteria were more abundant in AD subjects, while Cyanobacteria, Verrucromicrobia and Tenericutes were more abundant in controls. In addition, the relative abundance of two classes was higher in AD subjects (Gammaproteobacteria and Fusobacteria), and 5 classes were more abundant in controls (Clostridia, Verrocomicrobiae, Mollicutes, Erysipelotrichiae and Melainabacteria). Finally, relative abundance of 20/198 genera was significantly higher in controls (Figure 3), including 4 with LDA score >3.5 (*Christensenellaceae R-7*, *Ruminococcaceae UCG-014*, *Akkermansia* and *[Eubacterium] eligens group*); while 6/198 genera were significantly more abundant in the AD group, including 2 with LDA > 3.5 (*Escherichia-Shigella* and *Megasphaera*) (Figure 4). Afterwards, 23 of the 26 genera differentiated by LEfSe were analyzed with MaAsLin2. After adjusting for age, sex and BMI, 12 genera were significantly more abundant in controls and 3 were more abundant in AD. Notably, all associated genera with LDA score > 3.5 were concordant in the MaAsLin2 analysis (*p* ≤ 0.01, q ≤ 0.1), except for *Akkermansia*, as its association lost significance after adjusting for confounders (*p* = 0.156, *q* = 0.335; Supplementary Table S2).

**Figure 5:**
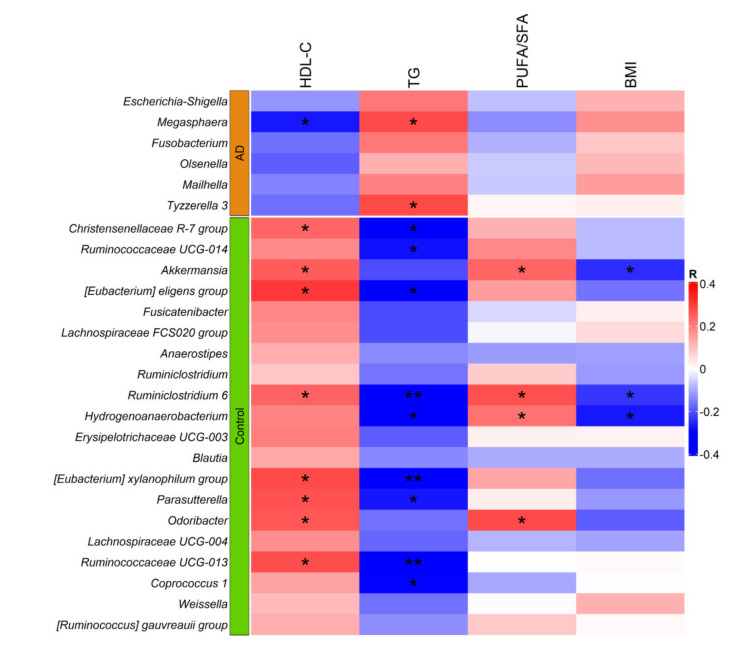
Heatmap showing correlations of relative genera abundance with HDL-C, TG, PUFA/SFA ratio, and BMI. HDL-C, high density lipoprotein cholesterol; TG, triglycerides; PUFA, polyunsaturated fatty acids; SAF, saturated fatty acids and BMI, body mass index. * *p* < 0.05; ** *p* < 0.005.

The relative abundance of these 26 genera was then tested for correlations with HDL-C and TG levels in the entire sample (AD patients and controls). As expected, all 6 genera found to be more abundant in subjects with AD correlated negatively with HDL-C levels and positively with TG levels, although only the correlations of *Megasphaera* genus abundance with both lipid parameters were significant. Similarly, all 20 genera more abundant in controls correlated positively with HDL-C and negatively with TG levels. Six of these genera correlated significantly with both lipid parameters (*p* < 0.05). Notably, the negative correlations of *Ruminococcaceae UCG-013*, *[Eubacterium] xylanophilum group*, *Ruminiclostridium 6*, *[Eubacterium] eligens group* amd *Christensenellaceae R-7 group* with TG levels remained significant after correcting for multiple tests (q < 0.05). Moreover, 4 microbial genera found to be more abundant in controls (*Akkermansia*, *Ruminiclostridium-6*, *Hydrogenoanearobacterium* and *Odoribacter*), showed a positive correlation with PUFA/SFA ratio (*p* < 0.05; q < 0.2). Notably, only the correlation with *Odoribacter* remained significant after adjusting for BMI (*p* = 0.025), consistent with the negative and significant correlation of *Akkermansia*, *Ruminiclostridium-6*, and *Hydrogenoanearobacterium* abundances with BMI (Figure 5).

### 3.4. Differences in Microbiota Functional Profiles

**Figure 6:**
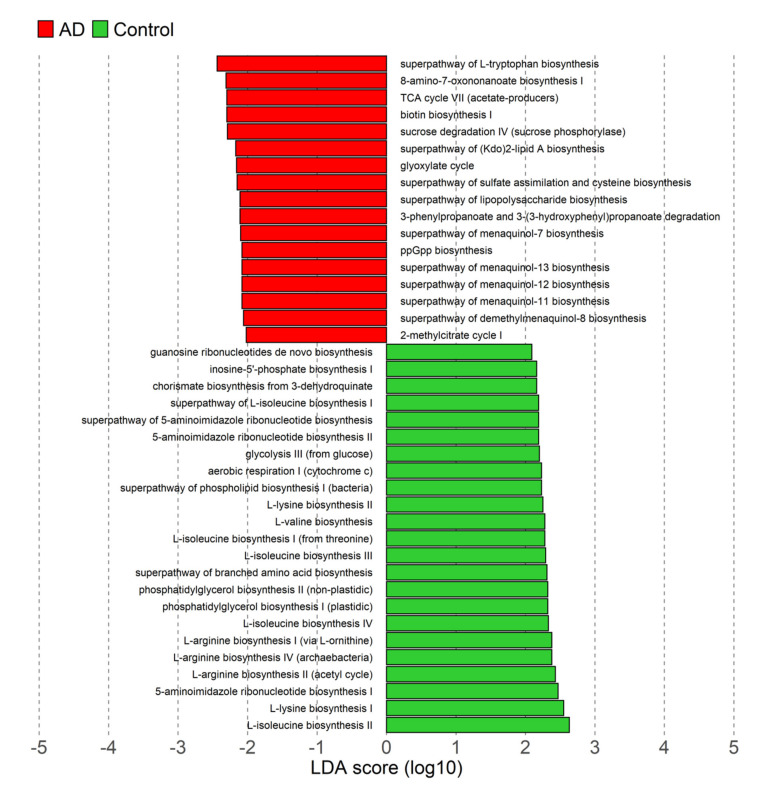
Pathway enrichment analyses and functional categories in AD cases. Pathways and functional categories with LDA score > 2 and *p* < 0.05 are shown.

To further investigate the potential mechanistic links between the gut microbiota, diet components and AD, a predictive functional analysis was performed. We identified 40 differentiated microbial MetaCyc metabolic pathways between AD and control groups. Overall, 17 pathways were found to be enriched in atherogenic dyslipidemia, including lipopolysaccharide and Kdo2-Lipid A biosynthesis (Figure 6). Moreover, the relative abundance of *Escherichia-Shigella* showed a strong and positive correlation with LPS (Rho = 0.984; *p* = 2.3 × 10^−59^) and Kdo2-Lipid A (Rho = 0.787; *p* = 8.0 × 10^−18^) biosynthesis pathways.

### 3.5. Gut Microbiota Associated with TG and HDL-C Levels before and after RYGB Surgery

In patients with RYGB, TG levels decreased while HDL-C levels increased significantly 12 months after surgery (*p* < 0.01; Table 2).

**Figure 7:**
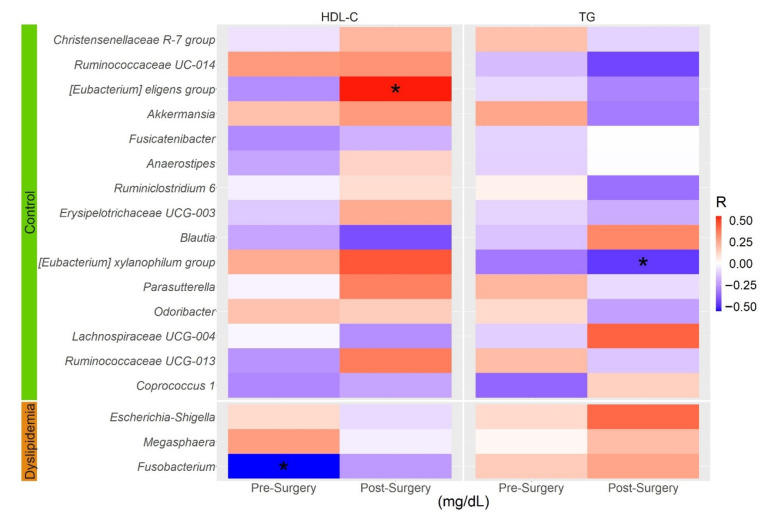
Heatmap of Spearman’s pairwise correlation coefficients between bacterial genera identified by LEfSe and lipid levels. Blue squares indicate negative correlations, and red squares indicate positive correlations. BMI, body mass index; HDL-C, high density lipoprotein-cholesterol; TG, triglycerides * *p* < 0.05.

**Figure 8:**
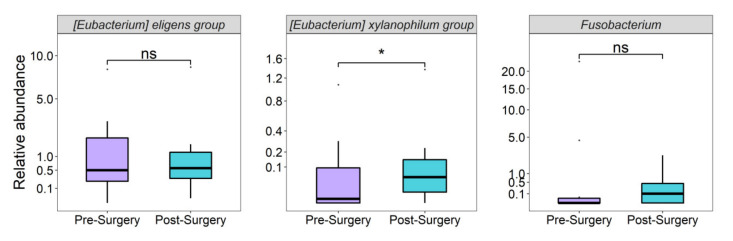
Relative abundance of *[Eubacterium] eligens group*, *[Eubacterium] xylanophilum group* and *Fusobacterium* in patients with AD before and after bariatric surgery. * *p* < 0.05, ns; not significant.

We thus sought correlations of the genera differentiated between AD patients and controls found in at least 25% of RYGB participants (18 of the 26 genera), with HDL-C and TG levels in these patients. Before surgery, only a negative and significant correlation between *Fusobacterium* relative abundance and HDL-C levels was observed, consistent with the higher abundance of this genus in AD patients (Figure 7). After surgery, two significant correlations were observed: a positive correlation of *[Eubacterium] eligens group* with HDL-C levels, and a negative correlation of *[Eubacterium] xylanophylum group* with TG levels (Figure 5), which remained significant after adjusting for BMI (*p* < 0.05). Only 10 of the 20 bariatric surgery patients met the diagnostic criteria for AD, whose lipid profiles improved significantly after surgery (Supplementary Table S3). We then compared the relative abundance of these three genera before and after surgery in these 10 patients. Only the relative abundance of *[Eubacterium] xylanophilum group* increased significantly after bariatric surgery in AD subjects (*p* = 0.03; Figure 8), consistent with the positive and negative correlations of this genus with HDL-C and TG levels, respectively, in both study groups (AD cases/controls and bariatric surgery).

## 4. Discussion

In this study, we found that gut microbiota diversity and abundance were significantly associated with atherogenic dyslipidemia. Decreased alpha diversity was observed in subjects with atherogenic dyslipidemia, in consistency with previous studies in European and Asian populations reporting a negative correlation between gut microbiota diversity and TG levels, and positive correlation with HDL-C levels [12,16,45].

The association of lower abundance of three genera (*Coprococcus 1*, *Christensenellaceae R-7* and *Odoribacter*) with atherogenic dyslipidemia found in the present study is in line with a previous report in a European populaion from the LifeLines-DEEP cohort [12]. In this cohort, *Coprococcus* abundance showed a strong and negative association with triglyceride levels (*p* = 6 × 10^−5^) [12]. *Coprococcus* species are SCFA-producing bacteria, known to decrease lipogenesis and to improve insulin resistance [46]. A recent study reported that omega-3 polyunsaturated fatty acid supplementation increases *Coprococcus* abundance, positively associated with serum levels of SCFA and branched-chain fatty acids, and negatively associated with triglyceride levels [47]. These findings suggest that the cardiovascular benefits associated with a higher PUFA intake may be at least partially mediated by the gut microbiome. However, in the present study *Coprococcus* abundance was not significantly associated with PUFA/SFA ratio. Moreover, *Christensenellaceae* abundance was associated with lower triglyceride levels (*p* = 2 × 10^−5^) and higher HDL-C levels (*p* = 0.004) in Europeans. *Christensenellaceae* has been inversely related to host body mass index (BMI) in different populations including Mexican children [48,49]. Because increased BMI is associated with dyslipidemia, an inverse association of *Christensenellaceae R-7 group* abundance with dyslipidemia dependent of BMI would be expected [50]. However, we observed that the association *Christensenellaceae* with AD remained significant after adjusting for BMI, suggesting that this association could be independent of BMI. Nevertheless, the mechanism underlying its negative association with atherogenic dyslipidemia remains to be elucidated. Finally, *Odoribacter* genus was associated with lower triglyceride levels in the LifeLines-DEEP cohort (*p* = 0.001) [12]. *Odoribacter* was also found to be associated with a healthy fasting serum lipid profile in European women with obesity [51]. In our study, the association of *Odoribacter* with a healthy lipid profile lost significance after adjusting for BMI. This is consistent with a previous report, where *Odoribacter* abundance was inversely associated with obesity in Mexican children [49]. Interestingly, *Odoribacter*, a SCFA-producing bacterium, has been associated with a healthy fasting serum lipid profile jointly with the SCFA-producing *Akkermansia* [12,51]. Thus, their possible SCFA-mediated role in metabolic disorders such as dyslipidemia, could be BMI dependent.

Notably, we observed higher abundance of *Eubacterium* (*eligens* and *xylanophilum*), *Ruminococcaceae*, *Ruminiclostridium* and *Blautia* genera in controls. All these genera are known to produce different types of SCFAs [52]. Although functional prediction of gut microbiota profiles in controls did not identify enrichment of any metabolic pathway directly related with SCFA biosynthesis, our results suggest that SCFA-producing bacteria may confer protection against atherogenic dyslipidemia.

In contrast with the control group findings, all genera with increased abundance in AD subjects are not known SCFA-producing bacteria. AD patients showed gut microbiota dysbiosis characterized by an overall reduction in microbial richness and diversity as compared to control subjects. Moreover, 6 genera showed higher abundance in this group, *Escherichia-Shigella* with the highest LDA score. In line with our findings, *Escherichia coli* has been associated with metabolic traits such as higher triglyceride levels [53] and non-alcoholic fatty liver disease (NAFLD) [54]. It has been suggested that *Escherichia-Shigella* increases intestinal permeability in humans by elevating LPS levels in the gut lumen [55,56]. This is in line with our functional prediction results showing enriched lipopolysaccharide and Kdo2-Lipid A biosynthesis pathways in atherogenic dyslipidemia patients, although bacterial gene expression studies are required to validate the role of these pathways in AD. In animal models, LPS administration produced hypertriglyceridemia by increasing hepatic fatty acid synthesis and adipose tissue lipolysis, while suppressing fatty acid oxidation [57]. Interestingly, increased *Escherichia-Shigella* abundance has been observed following RYGB surgery, with no apparent detrimental effects on the host [58]. However, in an independent study, *Escherichia-Shigella* abundance was significantly correlated with increased LDL-cholesterol levels 3 months after RYGB surgery [23]. In our study, relative *Escherichia-Shigella* abundance increased significantly after RYGB and correlated positively with triglyceride levels and negatively with HDL-C levels, without reaching statistical significance. Further studies with a longer follow-up are needed to assess whether the increased abundance of this genus is in fact metabolically detrimental to the host.

*Fusobacterium* abundance was also significantly increased in the atherogenic dyslipidemia group and showed a negative correlation with HDL-C levels before bariatric surgery. This is consistent with previous studies reporting higher abundance of this genus in subjects with metabolic unhealthy obesity, hypertriglyceridemia and T2D [16,45,59]. Although the mechanism is unclear, several studies suggest that genera highly abundant in metabolic disorders such as *Fusobacterium* are involved in inflammatory processes, possibly altering gut barrier permeability [60].

Of all macronutrients assessed in the FFQ, PUFA intake and PUFA/SFA ratio were significantly lower AD cases than in controls, as previously described for dyslipidemia and other metabolic diseases [61]. Although dietary factors are known to modulate gut microbiota composition, only four of the 26 genera were significantly associated with PUFA/SFA intake ratio: *Akkermansia*, *Ruminiclostridium 6*, *Hydrogenoanaerobacterium* and *Odoribacter*. This suggests that external factors other than diet, and host-related factors such as genetic variation, also can modulate gut microbiota composition [18]. In this regard, *Christensenellaceae*, *Odoribacter* and Tenericutes phylum abundance, which was increased in controls, has been found to be highly heritable across multiple populations [48,62]. Because the Mexican population has a high prevalence of atherogenic dyslipidemia, and a particular genetic architecture conferring increased risk of dyslipidemia, studies analyzing the relationships of genetic variation, microbiota composition and dyslipidemia are required in this population.

Several studies have reported that RYGB causes significant changes in microbiota composition [63]. In the present study, one year after surgery only 2 SCFA-producing genera, which were decreased in AD patients, were significantly associated with lipid levels: *[Eubacterium] eligens group* with higher HDL-C levels and *[Eubacterium] xylanophilum group* with lower triglyceride levels. In a previous study, *[Eubacterium] eligens group* showed a strong and negative correlation with visceral abdominal fat area and triglyceride levels, and a positive correlation with HDL-C levels [64]. Likewise, *[Eubacterium] xylanophilum group* showed a negative association with body weight and total serum cholesterol levels [65]. *[Eubacterium] xylanophilum* is a potent butyrate-producing bacterium in the gut. Butyrate increases after RYGB surgery in humans and murine models [66]. It has been suggested that the beneficial effect of butyrate on diet-induced obesity and atherosclerosis risk is mediated by the regulation of the expression of genes involved in lipid and glucose metabolism [67,68]. Although the abundance of these genera increased after surgery in our AD patients, only *[Eubacterium] xylanophilum* was significantly more abundant. Whether these SCFA-producing genera play a relevant role in lipid profile improvement after RYGB surgery requires further study.

Some limitations of the study should be acknowledged. Firstly, this was a cross-sectional study, not allowing to establish a causal relationship between the identified genera and AD. The associations were adjusted for confounders such as BMI, but not for other microbiota modulators such as statin use [69]. Because we used 16rRNA gene sequencing, bacterial metagenome sequencing is required to better identify the species and metabolic pathways associated with atherogenic dyslipidemia. Moreover, gut microbiota metabolites with a possible role in dyslipidemia-related mechanisms such as SCFA and LPS were not measured, and PUFA/SFA intake was not determined in subjects with obesity undergoing bariatric surgery. Finally, as we only included 20 RYGB patients, the associations with lipid profiles found in RYGB patients need to be confirmed in larger cohorts.

## 5. Conclusions

In conclusion, SCFA-producing genera were significantly more abundant in the control group without atherogenic dyslipidemia, and some were associated with a better lipid profile in RYGB patients 12 months after surgery. The significantly lower diversity of the gut microbiota observed in atherogenic dyslipidemia patients was accompanied by increased abundance of potentially pathogenic LPS-producing bacteria such as Escherichia-shigella. To our knowledge, this is the first study assessing the association of gut microbiota with atherogenic dyslipidemia, and its impact on serum lipid levels after bariatric surgery.

## Figures and Tables

**Table 1:** Comparison of anthropometric and biochemical characteristics in atherogenic dyslipidemia subjects and controls.

Trait	Atherogenic Dyslipidemia	Control	*p*
	(*n* = 41)	(*n* = 38)	
Female, *n* (%)	31 (75.6)	32 (84.2)	0.342
Age, years	59.0 (48.0–69.5)	55.0 (35.0–64.0)	0.160
BMI, kg/m^2^	27.9 (26.3–30.7)	24.1 (21.9–26.9)	1.0 × 10^−5^
HDL-C, mg/dL	38.0 (33.0–41.8)	67.9 (61.9–73.9)	2.1 × 10^−14^
Triglycerides, mg/dL	229.0 (183.5–267.5)	90.0 (71.3–108.0)	2.1 × 10^−14^
Non HDL-C, mg/dL	139.0 (122.6–163.5)	137.7 (115.9–160.9)	0.312
Total cholesterol, mg/dL	179.0 (161.0–197.5)	208.0 (180.0–229.3)	0.004
Fasting glucose, mg/dL	99.0 (92.5–109.5)	92.5 (85.8–97.3)	0.002
Diabetes, *n* (%)	6 (14.6)	3 (7.9)	0.207
Hypolipidemic treatment, *n* (%)	9 (22.0)	0 (0)	0.002

Data are presented as median (interquartile range) or as number (percentage). BMI, Body mass index; HDL-C, High density lipoprotein cholesterol.

**Table 2:** Comparison of anthropometric and biochemical parameters before and after bariatric surgery.

Trait	Pre-Surgery	Post-Surgery	*p*
	(*n* = 20)	(*n* = 20)	
Female, *n* (%)	13 (65.0)	-	-
Age, years	40.0 (31.3–44.8)	-	-
BMI, kg/m^2^	45.7 (42.3–51.9)	32.9 (28.7–36.2)	5.0 × 10^−6^
HDL-C, mg/dL	35.0 (31.3–41.8)	45.0 (37.0–49.0)	0.001
Triglycerides, mg/dL	159.5 (104.3–180.8)	100.0 (67.0–144.0)	0.011
Total cholesterol, mg/dL	154.5 (136.3–186.5)	86.0 (78.0–96.0)	0.097
Hypolipidemic treatment, *n* (%)	4 (20.0)	1 (5.0)	0.151

Data are presented as median (interquartile range) or as number (percentage). BMI, Body mass index; HDL-C, High density lipoprotein cholesterol.

## Data Availability

Data supporting the findings of this study are available on reasonable request.

## Supplementary Materials

The following supporting information can be downloaded at: https://www.mdpi.com/2072-6643/14/17/3545/s1, Table S1: Comparison of dietary macronutrient intake in AD cases and controls; Figure S1: Comparison of dietary macronutrient intake in AD cases and controls; Figure S2: LEfSe plot showing differentially abundant phyla between controls and atherogenic dyslipidemia subjects (AD); Table S2: Association of genera abundance adjusting for age, sex and BMI; Figure S3: Pathway enrichment analyses and functional categories in AD cases and controls.
