# Supplementary material for: Association of Gut Microbiota with Atherogenic Dyslipidemia, and Its Impact on Serum Lipid Levels after Bariatric Surgery
Source: Nutrients. 2022 Aug 28;14(17):3545. doi: 10.3390/nu14173545 (PMC9460232; doi:10.3390/nu14173545)
Supplement: Supplementary file 1 [file nutrients-14-03545-s001.zip › nutrients-1858743-supplementary.pdf]

Supplementary Materials:

Association of gut microbiota with atherogenic dyslipidemia, and its impact on serum lipid levels after bariatric surgery

Table S1. Comparison of dietary macronutrient intake in AD cases and controls.

**Trait**	**Atherogenic dyslipidemia** (n=41)	**Control** (n=38)	***P***
Energy, kcal/day	1556.90 (1221.88 – 1994.62)	1595.68 (1278.28 – 2290.65)	0.841
Carbohydrates (%)	61.94 (56.84 – 66.55)	59.31 (53.27 – 67.92)	0.399
Protein (%)	13.77 (11.98 – 15.05)	13.75 (12.06 – 15.35)	0.746
Total fat (%)	24.40 (20.70 – 28.83)	26.91 (20.40 – 31.82)	0.280
SFA (%)	8.09 (6.84 – 10.48)	8.81 (6.13 – 9.96)	0.631
MUFA (%)	10.85 (8.43 – 12.36)	11.91 (8.69 – 14.55)	0.164
PUFA (%)	4.92 (4.18 – 6.18)	5.75 (4.61 – 7.11)	0.035
Dietary fiber (g/1000kcal)	15.18 (10.42 – 20.42)	17.24 (11.14 – 21.65)	0.524

Data are presented as median (interquartile range). P-values were obtained using Mann-Whitney U-test. AD, atherogenic dyslipidemia; SFA, saturated fatty acids; MUFA, monounsaturated fatty acids; PUFA, polyunsaturated fatty acids.

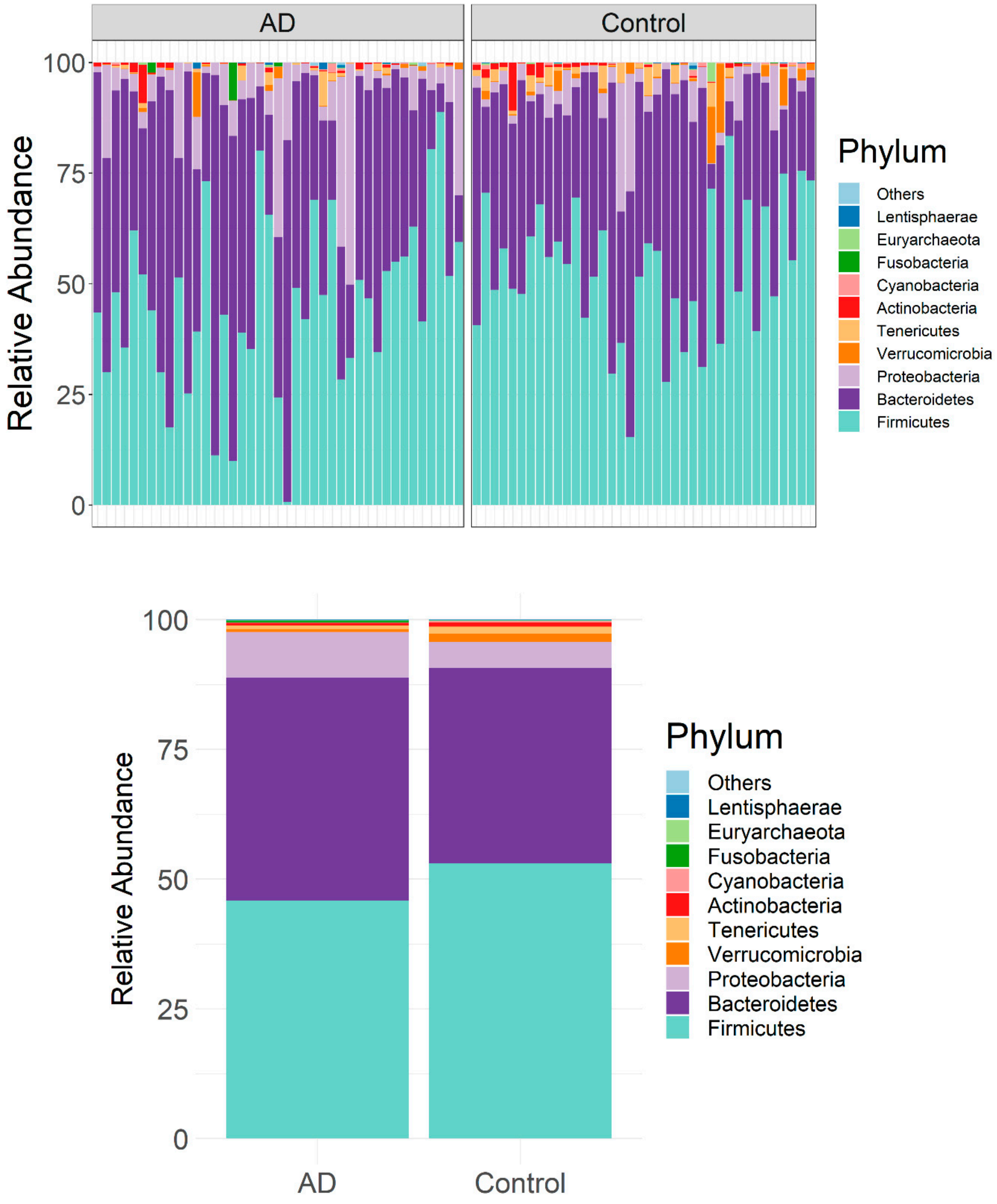

Figure S1. Distribution of bacterial composition at phylum level in atherogenic dyslipidemia (AD) patients and controls.

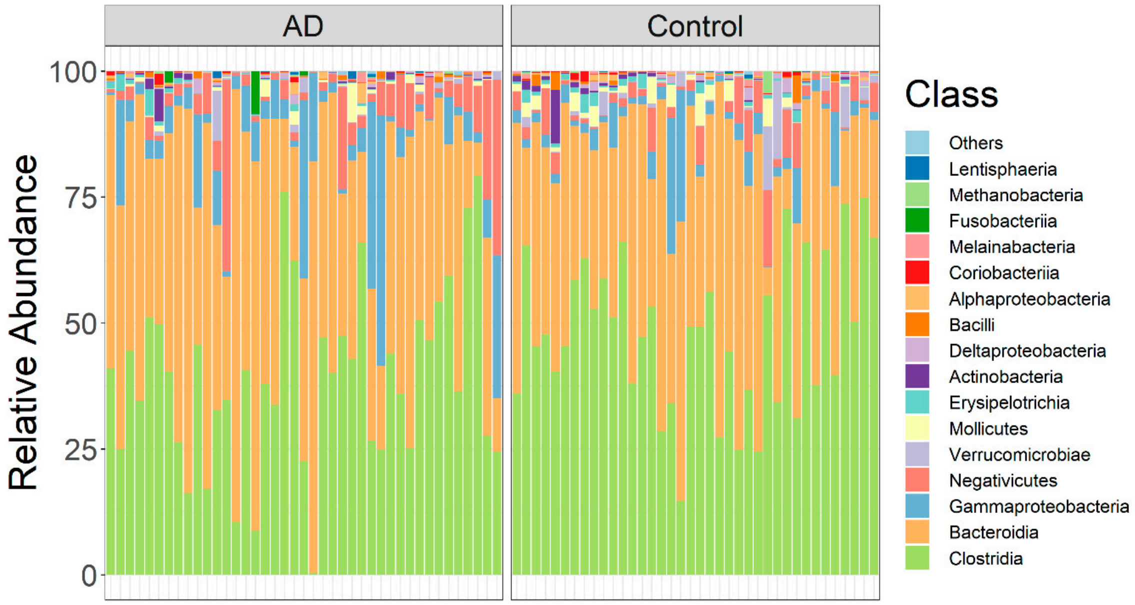

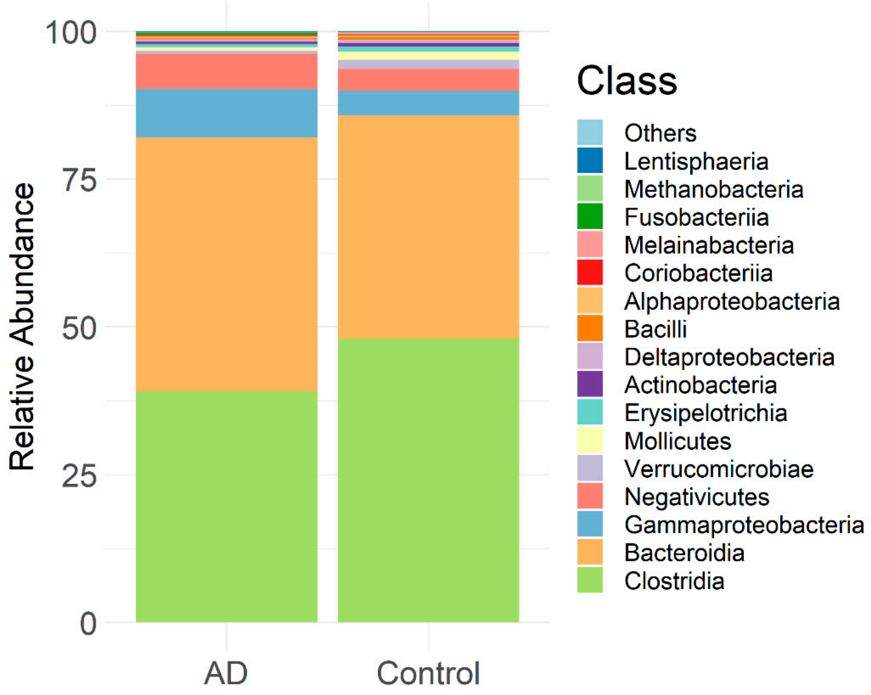

Figure S2. Distribution of bacterial composition at the class level in atherogenic dyslipidemia (AD) patients and controls.

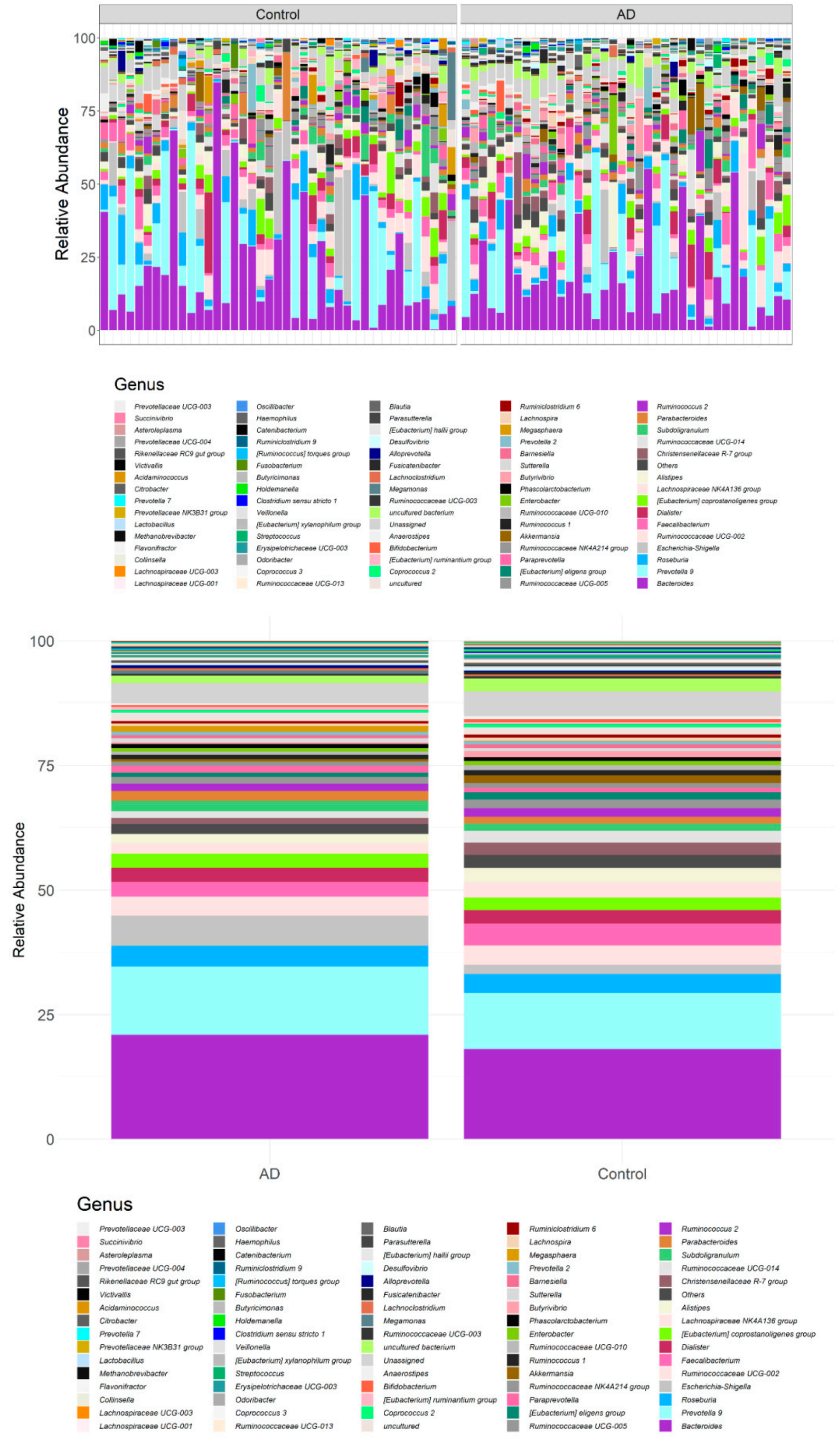

Figure S3. Distribution of bacterial composition at the genus levels in atherogenic dyslipidemia (AD) patients and controls.

Table S2. Association of genera abundance with atherogenic dyslipidemia adjusting for age, sex and BMI.

Genus	Coefficients	*P*-value	*q*-value
[Eubacterium] eligens group	-2.005	0.002	0.045
Parasutterella	-2.394	0.003	0.046
Lachnospiraceae FCS020 group	-0.795	0.004	0.046
Ruminococcaceae UCG 014	-2.175	0.008	0.080
[Eubacterium] xylanophilum.group	-1.661	0.007	0.080
Escherichia-Shigella	1.753	0.010	0.082
Fusicatenibacter	-1.527	0.010	0.082
Christensenellaceae R 7 group	-2.091	0.013	0.086
Erysipelotrichaceae UCG 003	-1.725	0.014	0.086
Megasphaera	2.128	0.022	0.111
Olsenella	0.606	0.026	0.125
Ruminiclostridium 6	-1.855	0.033	0.139
Lachnospiraceae UCG 004	-1.157	0.033	0.139
Ruminococcaceae UCG 013	-1.399	0.038	0.152
Coprococcus 1	-1.057	0.042	0.156
Fusobacterium	1.206	0.051	0.178
[Ruminococcus] gauvreauii.group	-0.585	0.087	0.253
Anaerostipes	-1.313	0.097	0.263
Odoribacter	-0.734	0.116	0.304
Akkermansia	-1.294	0.157	0.336
Hydrogenoanaerobacterium	-0.379	0.221	0.414
Weissella	-0.341	0.239	0.423
Blautia	-0.628	0.349	0.573

Coefficients form the generalized linear model using MaAsLin2 on pairwise testing between the atherogenic dyslipidemia and the control groups. *q*-values were calculated using FDR correction.

Table S3. Comparison of anthropometric and biochemical parameters of atherogenic dyslipidemia (AD) patients before and after bariatric surgery.

Trait	AD subjects (n=10)		
	**Pre-surgery**	**Post-surgery**	***P***
Female, n (%)	7 (70)	-	-
Age, years	41.5 (30.3 – 51.0)	-	-
BMI, kg/m^2	48.3 (41.3 – 55.7)	32.9 (28.8 – 37.7)	0.002
HDL-C, mg/dL	33.0 (30.8 – 36.5)	42.0 (37.0 – 46.5)	0.010
Triglycerides, mg/dL	175.0 (162.8 – 183.3)	135.0 (108.3 – 149.0)	0.002
Total cholesterol, mg/dL	157.0 (133.8 – 189.3)	143.0 (124.5 – 159.8)	0.257
Hypolipidemic treatment, n (%)	3 (30)	1 (10)	0.264

Data are presented as median (interquartile range) or as number and percentage. BMI, Body mass index; HDL-C, High density lipoprotein cholesterol.
